# Lipopolysaccharide pretreatment inhibits LPS-induced human umbilical cord mesenchymal stem cell apoptosis via upregulating the expression of cellular FLICE-inhibitory protein

**DOI:** 10.3892/mmr.2015.3723

**Published:** 2015-05-04

**Authors:** YU SEN HOU, LING YING LIU, JIA KE CHAI, YONG HUI YU, HONG JIE DUAN, QUAN HU, HUI NAN YIN, YI HE WANG, SHU BO ZHUANG, JUN FAN, WAN LI CHU, LI MA

**Affiliations:** 1 Department of Burns and Plastic Surgery, The First Affiliated Hospital of PLA General Hospital, Beijing 100048, P.R. China; 2 Beijing Fengtai You’an Men Hospital, Beijing 100069, P.R. China

**Keywords:** human umbilical cord mesenchymal stem cells, lipopolysaccharide, pretreatment, cFADD-like IL-1β-converting enzyme inhibitory protein, apoptosis

## Abstract

Mesenchymal stem cell (MSC)-based regenerative therapy is currently regarded as a novel approach with which to repair damaged tissues. However, the efficiency of MSC transplantation is limited due to the low survival rate of engrafted MSCs. Lipopolysaccharide (LPS) production is increased in numerous diseases and serves an essential function in the regulation of apoptosis in a variety of cell types. Previous studies have indicated that low-dose LPS pretreatment contributes to cytoprotection. In the current study, LPS was demonstrated to induce apoptosis in human umbilical cord mesenchymal stem cells (hUCMSCs) via the activation of caspase, in a dose-dependent manner. Low-dose LPS pretreatment may protect hUCMSCs against apoptosis induced by high-dose LPS, by upregulating the expression of cellular FADD-like IL-1β-converting enzyme-inhibitory protein (c-FLIP). The results of the present study indicate that pretreatment with an appropriate concentration of LPS may alleviate high-dose LPS-induced apoptosis.

## Introduction

Mesenchymal stem cells (MSCs) are non-hematopoietic multi-potent stem cells that possess therapeutic potential due to their capacity for self-renewal, expansion and differentiation, in addition to the ease of isolation from bone marrow, the umbilical cord and other tissues (1–4). Human umbilical cord MSCs (hUCMSCs) belong to a population of stem cells (5) that are used to treat certain intractable diseases (6,7). Experimental and clinical studies have indicated that transplantation with MSCs may be a promising strategy for the regeneration and repair of damaged tissues (4,8). However, the primary challenge in stem cell therapy remains the need to reduce the apoptosis of these cells, which occurs when exposed to harsh, proapoptotic microenvironments, such as the presence of excessive inflammatory stimuli, oxidative stress or cytotoxic free radicals (9,10). Therefore, it is important to identify the factors that are involved in the induction of apoptosis of MSCs, as well as those which promote their survival.

Lipopolysaccharide (LPS), a component of the outer membrane of gram-negative bacteria, is an important mediator of endotoxemia, multiple organ dysfunction syndrome and endotoxic shock. These may occur in patients as a result of sepsis, severe burns or trauma (11,12). In addition to stimulating innate immunity via regulation of the production of inflammatory mediators, LPS may also result in apoptosis and cell death in multiple cell types, including macrophages (13), endothelial cells (14) and microglial cells (15). However, other studies have suggested that an appropriate concentration of LPS pretreatment may protect neurons (16,17), myocardial cells (18) and bone marrow stem cells (19) from apoptosis induced by ischemic injury or oxidative stress. Although LPS pretreatment has been extensively investigated, little is known regarding the effect of pretreatment with LPS on endotoxin-induced apoptosis in MSCs.

It is well-established that apoptosis is a tightly regulated process (20). One of the primary proteins responsible for regulating apoptosis is the cellular FADD-like IL-1β-converting enzyme (FLICE)-inhibitory protein (c-FLIP), which was originally identified as an inhibitor of caspase 8. In addition to the full-length form of c-FLIP (55 kDa), also termed p55-FLIP or c-FLIP_L_, which contains two death effector domains (DED) and a caspase-like domain, two proteolytically processed forms of c-FLIP_L_ have been characterized, including p43-FLIP (43 kDa) and the shorter, p22-FLIP (22 kDa), which contain the two DED domains without the caspase-like domain (21). c-FLIP binds to either Fas-associated protein with death domain (FADD) or caspase 8, through DED-DED interactions, thus inhibiting the activation of caspase 8 (22). A number of studies have demonstrated that LPS and tumor necrosis factor-α (TNF-α) upregulate c-FLIP and suppress Fas-FasL mediated caspase 8 and 3 activation, as well as cell apoptosis (23,24).

The present study was designed to investigate whether repetitive exposure to LPS protects hUCMSCs against the apoptotic consequences of subsequent endoxin insults, and whether LPS-induced adaptive cytoprotection is associated with overexpression of the c-FLIP protein.

## Materials and methods

### Materials

Dulbecco’s modified Eagle’s medium (DMEM)/F12, trypsin, fetal bovine serum (FBS) and type-II collagenase were purchased from Gibco Life Technologies (Carlsbad, CA, USA); penicillin-streptomycin was from GE Healthcare Bio-Sciences (Pittsburgh, PA, USA); lipopolysaccharides (*Escherichia coli* serotype 055:B5) and 3-(4,5-dimethylthiazol-2-yl)-2,5-diphenyltetrazolium bromide (MTT) were from Sigma-Aldrich (St. Louis, MO, USA); Annexin V: FITC Apoptosis Detection kit was from BD Biosciences (San Diego, CA, USA); the Cell Death Detection ELISA^PLUS^ Assay kit was from Roche Diagnostics (Mannheim, Germany); anti-c-FLIP rabbit monoclonal antibody (#8510) was from Cell Signaling Technology, Inc. (Danvers, MA, USA); rabbit anti-GAPDH polyclonal antibody (bs-2188R) and goat anti-rabbit IgG polyclonal antibody (bs-0295G) were purchased from Bioss Company (Beijing, China); caspase 8 and 3 Activity Assay kits were purchased from Beyotime Institute of Biotechnology (Haimen, China).

### Preparation of hUCMSCs

hUCMSCs were isolated, as previously described (25). Umbilical cord tissues (15–20 cm) from three full-term healthy babies delivered by caesarean section at the First Affiliated Hospital of PLA General Hospital (Beijing, China), were thoroughly rinsed with phosphate-buffered saline (PBS) and cut into 1-mm^3^ samples, following removal of the umbilical vessels and external membrane. The tissues were placed in culture flasks (Corning, Tewksbury, MA, USA) at a distance of 0.5 cm with DMEM/F12, supplemented with 10% FBS and 1% penicillin/streptomycin at 37°C in a humidified atmosphere of 5% CO_2_. The medium was replaced slowly and gradually every 3 days, to ensure that fixation of the tissue. When cells in the tissue samples reached 80–85% confluence, the tissues were removed and the cells were digested with trypsin-EDTA (Gibco Life Technologies) and transferred to T-75 culture flasks for propagation and culture. Passage 3 cells were stored for use in subsequent studies. The protocol of the current study was approved by the ethics committee of the First Affiliated Hospital of PLA General Hospital (Beijing, China).

### Cell viability assay

hUCMSCs were inoculated in 96-well plates at a density of 2×10^3^ cells/well for 24 h, and the medium was replaced with media containing different concentrations of LPS (0, 0.01, 0.1, 1, 10, 20, 30, 40 or 50 *μ*g/ml) for continuous culturing. One plate from each concentration group was sampled at each time point (12, 24 and 48 h) and 20 *μ*l/well of 5 mg/ml MTT was added. Following a 4-h incubation, the supernatant was removed from the plate and dimethyl sulfoxide (200 *μ*l/well) was added. The samples were shaken for 5 min, the MTT formazan product was measured using a microplate reader (Synergy 2; BioTek Instruments, Ltd., Winooski, VT, USA) and the absorbance was measured at 490 nm.

### Flow cytometry analysis

hUCMSCs were cultured in 6-well plates and either treated with LPS (0, 1, 40 or 50 *μ*g/ml) for 24 h, or pretreated with LPS (0, 0.1, 1, 10 or 20 *μ*g/ml) for 12 h with subsequent exposure to 50 *μ*g/ml LPS for 24 h. Following treatment, cells were harvested using trypsin-EDTA and collected by centrifugation at 900 × g for 5 min at room temperature. Cells were washed, resuspended in PBS, and labeled with Annexin V and propidium iodide (PI; from the BD Apoptosis Detection kit) for 20 min. Analyses were performed by flow cytometry (FC500; Beckman Coulter, Brea, CA, USA) using FC500 MPL CXP2.1 software.

### Hoechst staining analysis

hUCMSCs were cultured in 6-well plates. Following treatment, cultured cells were washed twice with PBS and treated with 4% paraformaldehyde (Guge Biological Technology Co., Ltd., Wuhan, China) for 10 min. After three washes with PBS, the cells were stained with 5 *μ*g/ml Hoechst 33258 (Sigma-Aldrich) for 10 min. The morphology of the nuclei of the hUCMSCs was then analyzed under an inverted phase contrast microscope (DMI6000B; Leica Microsystems GmbH, Wetzlar, Germany).

### Western blot analysis

hUCMSCs, with or without LPS stimulation, were rinsed twice with ice-cold PBS and lysed with ice-cold lysis buffer [1% Triton X-100, 20 mmol/l HEPES (pH 7.5), 1 mmol/l each of EDTA, DTT and PMSF, and 1 mg/ml each of leupeptin, aprotinin and pepstatin; Macgene (Beijing) Biotechnology Ltd., Beijing, China] for 30 min. Cell and nuclear lysates were centrifuged at 12,000 × g for 10 min at 4°C, and the supernatants were mixed with 5X SDS sample buffer (Beijing Solarbio Science & Technology Co., Ltd., Beijing, China), boiled for 5 min and separated on 15% SDS-PAGE gels (Bio-Rad Laboratories, Inc., Hercules, CA, USA). Following electrophoresis, proteins were transferred to nitrocellulose blotting membranes by electrophoretic transfer. Non-specific binding was blocked with 5% non-fat milk for 1 h. The membrane (Merck Millipore, Darmstadt, Germany) was then rinsed with TBST and incubated overnight at 4°C with the primary antibody (c-FLIP, 1:1,000 or GAPDH, 1:1,500). It was then washed in TBST and incubated for 1 h with horseradish peroxidase-conjugated secondary antibody. After washing in TBST, bands were visualized using enhanced chemoluminescence and exposed to radiography film (Kodak, Tokyo, Japan).

### Silencing of c-FLIP expression with small interfering RNA (siRNA)

The targeting sequence of human c-FLIP is GGAGCAGGGACAAGTTACA (26). Double-stranded, siRNA for c-FLIP was chemically synthesized by GeneChem, Inc. (Shanghai, China). The transfection of siRNA was performed using Lipofectamine 2000 (Invitrogen Life Technologies, Carlsbad, CA, USA), according to the manufacturer’s instructions. In brief, cells were harvested using 0.25% trypsin, 1 mM EDTA in PBS without Ca^2+^ and Mg^2+^, and plated in 6-well plates at 10^5^ cells/cm^2^. Next, 5 *μ*l siRNA (100 lM) solution was mixed with 125 *μ*l serum-free culture medium for 5 min at room temperature. During this incubation period, 5 *μ*l Lipofectamine 2000 was diluted in 125 *μ*l serum-free culture medium. These two mixtures were combined, mixed gently, and incubated for 20 min at room temperature to allow complex formation. The resulting 250 *μ*l siRNA-Lipofectamine mixture was then added to the cells to produce a final volume of 2.5 ml/well. Forty-eight hours after infection, cells were washed and then incubated with LPS prior to use. Knockdown efficiencies were routinely monitored by western blotting.

### Analysis of caspase activity and internucleosomal degradation of genomic DNA

Caspase 3 and caspase 8 activity levels were measured with the Caspase-Glo Assay System (Promega Corporation, Madison, WI, USA), according to the manufacturer’s instructions. Internucleosomal degradation of genomic DNA was detected using the Cell Death Detection ELISA^PLUS^ assay. hUCMSCs that were either untreated or treated with LPS, with or without transfection of siRNA, were washed twice with cold PBS and lysed on ice in a cold lysis buffer. Cell lysates were centrifuged at 12,000 × g for 10 min in order to precipitate cellular debris. The assay was performed in a 96-well plate, according to the manufacturer’s instructions, using the microplate reader, and the absorbance was measured at 405 nm.

### Statistical analysis

All data are expressed as the mean ± standard deviation. Significance was analyzed using SPSS software, version 16.0 (SPSS Inc., Chicago, IL, USA). Statistical analysis was performed by one-way analysis of variance, followed by Bonferroni multiple-comparison test from >3 independent experiments. P<0.05 was considered to indicate a statistically significant difference.

## Results

### LPS induces apoptosis in hUCMSCs in a dose-dependent manner

**Figure 1:**
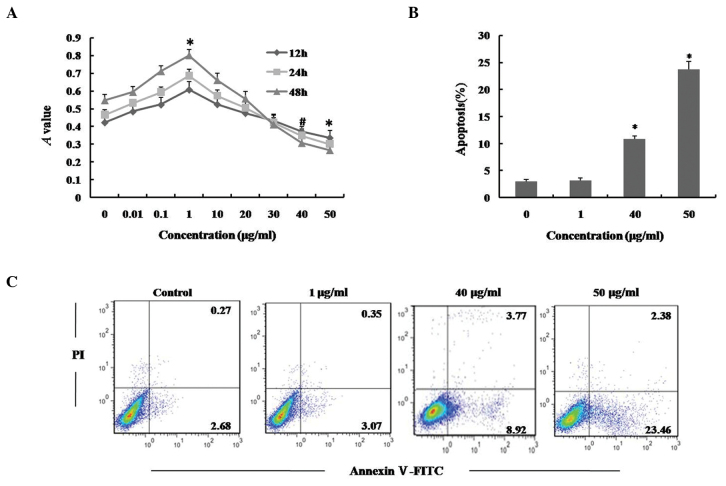
Effect of LPS on viability and apoptosis of hUCMSCs. (A) hUCMSCs were treated with 0, 0.01, 0.1, 1, 10, 20, 30, 40 or 50 *μ*g/ml LPS for 12, 24 and 48 h, and cell viability was determined using an MTT assay. (B) and (C) hUCMSCs were treated with 0, 1, 40 or 50 *μ*g/ml LPS for 24 h. The cells were stained with Annexin V/PI and apoptosis levels were determined using flow cytometry. Data are presented as the mean ± standard deviation. ^*^P<0.01, ^#^P<0.05 vs. control; n=5. LPS, lipopolysaccharide; hUCMSCs, human umbilical cord mesenchymal stem cells; PI, propidium iodide.

In order to investigate the cytotoxicity of LPS, hUCMSCs were incubated with various concentrations of LPS for 12, 24 and 48 h, and cell viability was examined using an MTT assay. As shown in Fig. 1A, exposure of hUCMSCs to 40 or 50 *μ*g/ml LPS resulted in a significant reduction in cell viability compared with a dose of 0 *μ*g/ml, for all three time periods, while the cell viability was increased following treatment with low concentrations of LPS. Flow cytometric analysis using Annexin V/PI staining, further confirmed that cells exposed to 40 and 50 *μ*g/ml LPS for 24 h exhibited a significant increase in apoptosis (Fig. 1B and C; P<0.01). By contrast, LPS (0.01–30 *μ*g/ml) produced little or no cytotoxicity in hUCMSCs, indicating that LPS may exert antithetical effects, depending upon its concentration; low doses of LPS appeared to enhance cell viability while higher doses exhibited cytotoxicity in hUCMSCs. The current data indicates that LPS induces apoptosis in hUCMSCs in a dose-dependent manner.

### Effects of pretreatment with different concentrations of LPS on LPS-induced apoptosis in hUCMSCs

**Figure 2:**
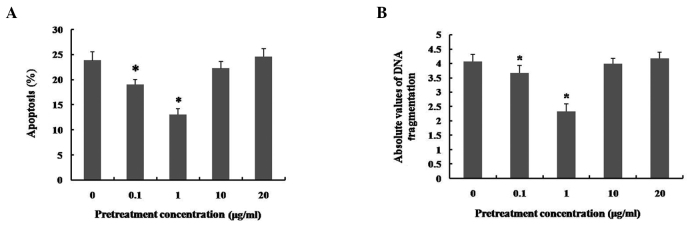
Effect of LPS pretreatment on high-dose LPS-induced apoptosis. hUCMSCs were pretreated with 0, 0.1, 1, 10 or 20 *μ*g/ml LPS for 12 h, with subsequent exposure to 50 *μ*g/ml LPS for 24 h. (A) The percentage of apoptotic hUCMSCs was analyzed by flow cytometry. (B) The severity of DNA fragmentation was analyzed using the Cell Death Detection ELISA^PLUS^ assay. Data are presented as the mean ± standard deviation. ^*^P<0.01 vs. control; n=5. LPS, lipopolysaccharide; hUCMSCs, human umbilical cord mesenchymal stem cells.

**Figure 3:**
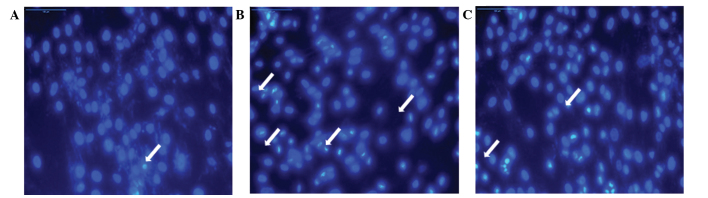
Nuclear staining to assess induction of hUCMSC apoptosis by LPS, and the cytoprotective capabilities of LPS pretreatment. Nuclear condensation was assessed using Hoechst 33258 for hUCMSCs. (A) Control, medium only. (B) hUCMSCs exposed to 50 *μ*g/ml LPS for 24 h exhibited marked apoptotic nuclear condensation. (C) hUCMSCs pretreated with 1 *μ*g/ml LPS for 12 h and then exposed to 50 *μ*g/ml LPS for 24 h did not exhibit nuclear condensation. hUCMSCs, human umbilical cord mesenchymal stem cells; LPS, lipopolysaccharide.

An increasing number of studies have reported that low-dose LPS pretreatment results in cytoprotection in several cell types (16–19). Fig. 1A also indicates that low doses of LPS may promote hUCMSC viability. In order to explore the possible cytoprotective effects of low doses of LPS, prior to administration of a high-dose of LPS (50 *μ*g/ml), LPS pretreatment was performed in hUCMSC cultures by incubation with a range of doses (0, 0.1, 1, 10 and 20 *μ*g/ml) of LPS for 12 h. The apoptosis induced by LPS at 50 *μ*g/ml was inhibited by pretreatment with 0.1 and 1 *μ*g/ml LPS (particularly with 1 *μ*g/ml; P<0.01). However, pretreatment with 10 and 20 *μ*g/ml LPS did not significantly alter the apoptosis induced by treatment with high-dose LPS (Fig. 2A). As indicated by the cell death detection ELISA^PLUS^ assay, the severity of DNA fragmentation in hUCMSCs was consistent with the flow cytometry results (Fig. 2B). In addition, the apoptotic nuclear condensation in hUCMSCs was detected by Hoechst 33258 staining. In addition, as shown by Hoechst 33258 staining, cells treated with 50 *μ*g/ml LPS presented apoptotic phenotypes, with clear chromatin conden sation, and cellular and nuclear shrinkage (Fig. 3A and B). LPS (1 *μ*g/ml) pretreatment markedly reduced 50 *μ*g/ml LPS-induced hUCMSC cell apoptosis (Fig. 3C). These results indicated that early exposure to low-dose LPS may protect hUCMSCs from the apoptotic consequences of subsequent lethal LPS insults, and that this effect is dependent upon the concentration of LPS used. The most effective cytoprotective effect was observed using 1 *μ*g/ml LPS, which had not effect on the induction of apoptosis in hUCMSCs. Therefore, this dose of LPS was selected for LPS pretreatment in the subsequent experiments.

### LPS pretreatment induces overexpression of c-FLIP

**Figure 4:**
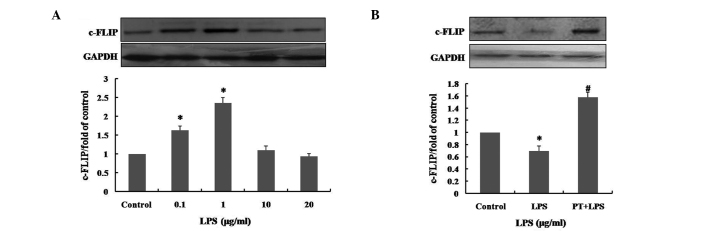
Effect of LPS pretreatment on c-FLIP expression levels in hUCMSCs. (A) Following pretreatment with 0, 0.1, 1, 10 or 20 *μ*g/ml LPS for 12 h, c-FLIP expression levels in hUCMSCs were determined by western blotting. (B) The c-FLIP expression levels, following exposure to 50 *μ*g/ml for 24 h with or without pretreatment with 1 *μ*g/ml LPS for 12 h were determined by western blotting. Control, medium only; LPS, 50 *μ*g/ml LPS treatment for 24 h; PT, 1 *μ*g/ml LPS pretreatment for 12 h. Data are presented as the mean ± standard deviation and are expressed as a percentage of the control value. ^*^P<0.01. vs. control and ^#^P<0.01, vs. LPS; n=5. LPS, lipopolysaccharide; c-FLIP, cellular FLICE-inhibitory protein; hUCMSCs, human umbilical cord mesenchymal stem cells.

c-FLIP has been demonstrated to be involved in the prevention of apoptosis in a number of systems (22,27). In order to explore whether pretreatment with low concentrations (0, 0.1, 1, 10 or 20 *μ*g/ml) of LPS modulates expression of c-FLIP in hUCMSCs, and to assess the response to high-dose LPS, the expression levels of c-FLIP were measured by western blot analysis. Following pretreatment with LPS for 12 h, the expression of c-FLIP was markedly increased in the hUCMSCs that had been pretreated with 0.1 and 1 *μ*g/ml LPS. Statistical analysis indicated that pretreatment with 0.1 and 1 *μ*g/ml LPS increased the expression level of c-FLIP to 1.63±0.11 and 2.36±0.15 times that of the control (P<0.01), respectively. However, pretreatment with 10 and 20 *μ*g/ml LPS did not significantly enhance c-FLIP expression levels (Fig. 4A).

Following exposure of hUCMSCs to 50 *μ*g/ml LPS for 24 h, the level of c-FLIP expression was markedly reduced, to 0.69±0.09-fold that of the control (P<0.01; Fig. 4B). However, the inhibition of the expression of c-FLIP induced by 50 *μ*g/ml LPS, was significantly reduced by pretreatment with 1 *μ*g/ml LPS for 12 h (P<0.01). This suggested that LPS pretreatment promotes the expression of c-FLIP and also prevents the reduction of expression induced by high-dose LPS treatment. It was thus inferred that the cytoprotective effects of pretreatment with low doses of LPS in hUCMSCs may be associated with overexpression of c-FLIP.

### LPS pretreatment prevents LPS-induced apoptosis by upregulating c-FLIP expression levels

**Figure 5:**
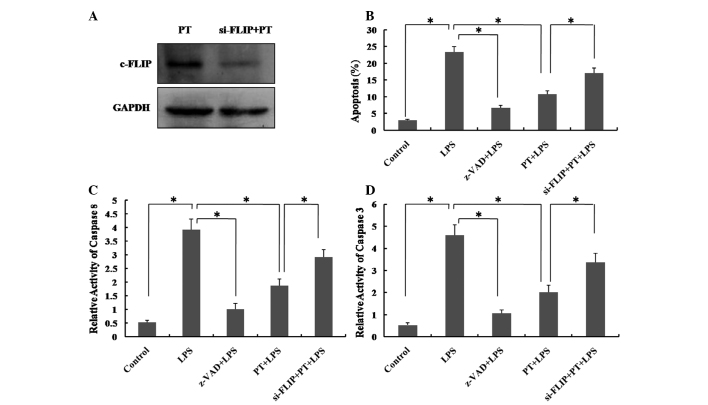
Effect of c-FLIP siRNA on LPS-induced apoptotic activity in hUCMSCs preconditioned with LPS was measured using flow cytometry, and caspase 8 and 3 ELISA assays. (A) Knockdown efficiency of c-FLIP was monitored by western blotting. (B) The percentage of apoptotic hUCMSCs was analyzed by flow cytometry. (C) and (D) The activity of caspase 8 and 3 was detected by ELISA. Control, medium only; LPS, hUCMSCs treated with LPS 50 *μ*g/ml for 24 h; Z-VAD, caspase inhibitor (20 *μ*M); PT, hUCMSCs preconditioned with LPS 1 *μ*g/ml for 12 h; si-FLIP, hUCMSCs transfected with siRNA of c-FLIP. Data are expressed as the mean ± standard deviation. ^*^P<0.01; n=5. c-FLIP, cellular FLICE-inhibitory protein; LPS, lipopolysaccharide; hUCMSCs, human umbilical cord mesenchymal stem cells; siRNA, small interfering RNA.

LPS has been used to induce a model of caspase-dependent apoptosis (26,28). As shown in Fig. 5, 50 *μ*g/ml LPS induced apoptosis, and enhanced the activity of caspase 8 and 3 in hUCMSCs, and these effects were inhibited by the caspase inhibitor Z-VAD-fmk. This indicates that LPS-mediated hUCMSC apoptosis is also caspase-dependent. c-FLIP has been shown to block the apoptotic pathway by interacting with caspase 8 at the death-inducing signaling complex, through DED-DED interactions (22,29). The present study aimed to determine whether the upregulation of c-FLIP induced by 1 *μ*g/ml LPS inhibits LPS-induced apoptosis in hUCMSCs that have been pretreated with low-dose LPS. Following treatment with 50 *μ*g/ml LPS for 24 h, the activity of caspase 8 and 3 in LPS-pretreated hUCMSCs was significantly reduced compared with that in the group without LPS pretreatment (Fig. 5C and D; P<0.01). In addition, following siRNA c-FLIP inhibition, caspase 8 and 3 activity, and the level of apoptosis in hUCMSCs subjected to high-dose LPS exposure, were significantly elevated (P<0.01), and this effect was reduced by pretreatment with 1 *μ*g/ml LPS for 12 h prior to exposure to the high-dose LPS. This result confirmed that LPS pretreatment prevents caspase-dependent apoptosis induced by high-dose LPS in hUCMSCs, via the upregulation of c-FLIP.

## Discussion

As a population of stem cells, hUCMSCs are able to self-renew, rapidly proliferate, differentiate and are involved in tissue repair and microenvironment regulation. These functions initially depend on the ability of cells to survive in a complex microenvironment. The findings of the present study indicate that LPS induces apoptosis in hUCMSCs by activating caspases in a dose-dependent manner, and that pretreatment with low concentrations of LPS markedly increases the survival of hUCMSCs under high-dose endotoxin conditions. In addition, the cytoprotective effect of LPS pretreatment against apoptosis induced by LPS in hUCMSCs is, in part, associated with the overexpression of c-FLIP.

As a result of bacterial infections and enterogenous endotoxin translocation, a large quantity of endotoxins is often present in patients who have received burns or other significant trauma (30). As a constitutive component of the bacterial cell wall in Gram-negative bacteria, LPS is an important mediator of endotoxemia and its invariant molecular pattern, lipid A, is specifically recognized by Toll-like receptor 4 (TLR4). TLR4s are expressed by MSCs and may be deleterious in MSC-mediated protection (31,32). TLR4 has been shown to induce apoptosis and inhibit proliferation in neuronal progenitor cells and various other cell types (13,15). A TLR4-mediated apoptotic pathway may be triggered by LPS and blocked by caspase inhibitors in endothelial cells (28). Other studies have demonstrated that LPS induces apoptosis in multiple cellular systems (15,28,33). The present study demonstrated that LPS induced apoptosis in hUCMSCs in a dose-dependent manner, indicating that a high level of endotoxins is a factor that influences hUCMSC survival. A hallmark of apoptosis is the activation of highly specific effector proteases of the caspase family. LPS has been reported to activate caspase 8 and 3 (34,35). Haase *et al* (13) also suggested that LPS/TLR4 induces apoptosis in macrophages via activation of caspase 8 and 3 in a FADD protein-dependent pathway (13). In the present study, it was shown that 50 *μ*g/ml LPS enhanced the activation of caspase 8 and 3, which is consistent with previous studies conducted in other cell systems (13,35).

Pretreatment creates a potent protective phenomenon in which tissues or cells develop resistance to the proapoptotic microenvironment, following exposure to a variety of injurious stimuli, including brief ischemia, hypoxia and low-dose LPS treatment (36–38). It has been shown that ischemic or oxidative pretreatment produces protective effects in the heart (39), brain (40,41) and kidneys (42). A number of studies have indicated that low-dose LPS pretreatment also elicits neuroprotection and protects MSCs from oxidative stress-induced apoptosis (16,43,44). The core principle of cytoprotection using pretreatment, is that the dose of the pretreatment stimulus should be high enough to produce an effect, while not causing cytotoxicity. The results of the current study indicated that repeated exposed to 0.1 and 1 *μ*g/ml LPS protected hUCMSCs against the apoptotic consequences of subsequent high-dose LPS insults, whereas 10 and 20 *μ*g/ml LPS did not. The reason for this may be that higher doses of LPS elicit deleterious effects. Although an increasing number of studies report a cytoprotective effect of LPS pretreatment, the molecular mechanism underlying the regulation of cytoprotection by LPS pretreatment remains unclear.

c-FLIP is a cytoplasmic protein, with sequence homology to FLICE. It binds to either FADD or caspase 8 via DED-DED interactions, resulting in inhibition of caspase 8 activation and apoptosis (45). The functional role of c-FLIP depends upon its level of expression; low levels of c-FLIP typically induce apoptosis while higher levels are cytoprotective (22,46). It has been demonstrated that LPS exposure rapidly upregulates the expression of c-FLIP in human dendritic cells (47). Other studies have reported that TNF-α and LPS induced the nuclear factor-κB-mediated expression of c-FLIP in a variety of cell types, promoting inducible resistance to death-receptor signaling (23,24). Using *in vitro* culture of hUCMSCs, the present study demonstrated that low concentrations of LPS enhance expression of c-FLIP, while high concentrations do not. Additionally, the results indicated that pretreatment with 1 *μ*g/ml LPS induced overexpression of c-FLIP and blocked high-dose LPS-induced inhibition of c-FLIP. It was hypothesized that overexpression of c-FLIP may be important for LPS pretreatment-mediated cytoprotection against high-dose LPS-induced apoptosis. In order to examine the association between the cytoprotection of LPS pretreatment and c-FLIP expression levels in hUCMSCs, c-FLIP siRNA was employed. The antiapoptotic effect of LPS pretreatment was weakened following the use of c-FLIP siRNA. This data confirmed that LPS pretreatment prevents the LPS-inducing caspase-dependent apoptosis in hUCMSCs through the induction of c-FLIP expression.

In conclusion, LPS induced apoptosis in hUCMSCs via activation of caspase in a dose-dependent manner. Pretreatment with low concentrations of LPS protected hUCMSCs against apoptosis induced by subsequent high-dose LPS insults. The cytoprotection effected by the LPS pretreatment occurred, in part, as a result of the overexpression of c-FLIP. However, the proapoptotic and antiapoptotic mechanisms are complex, and the antiapoptotic effect of LPS pretreatment may be associated with other unknown mechanisms. Additionally, the effect of LPS stimulation on the MSC phenotype and differentiation is unclear. Further investigations are required to address these remaining issues.
